# Editorial: Insights in emotion regulation and processing: 2022

**DOI:** 10.3389/fnbeh.2023.1271806

**Published:** 2023-09-29

**Authors:** Richard G. Hunter

**Affiliations:** Developmental and Brain Sciences Program, Department of Psychology, University of Massachusetts Boston, Boston, MA, United States

**Keywords:** emotion regulation, vagus, ADHD, mindfulness, working memory, gut-brain axis, major depression disorder, sensory regulation

The past year saw many new findings on the study of emotional regulation and processing. We have collected a few interesting findings in the area under the umbrella of “*Insights in emotion regulation and processing: 2022*”. The papers in the topic cover a broad range, as befits the complex array of influences that determine our ability to express and control our emotions. We include two empirical studies of human emotional regulation (Long et al.; Fan et al.), a review of the gut-brain axis in depression (Irum et al.) and two thoughtful pieces on cognitive reappraisal (Wang and Yin) and emotional and sensory regulation in attention deficit and hyperactivity disorder (ADHD) (Grossman and Avital).

In their contribution, Long et al. examine the hypothesis that non-emotional working memory (WM) training might improve adaptive emotional response, based on a number of recent findings relating high working memory to more adaptive emotional responding. They found that 15 day, but not 7 days of working memory training improved the degree of emotional responding to negative emotional pictures. These findings add to a growing literature that blurs the lines between “cold” cognitive processing and “hot” emotional processing systems and makes plain that both systems are intertwined in how we process our emotional experience. Also of interest is the finding that the WM training is dose responsive, suggesting longer term training may have even more substantial effects on our capacity to regulate our emotions effectively. Fan et al. examined the effects of a single mindfulness meditation on emotional event related potentials (ERP) in individuals with varying levels of fatigue. They found that the late positive potential was blunted in proportion to self-reported fatigue levels in the non-mindful control group, but not in the mindfulness group, suggesting that even a brief mindfulness intervention might improve responses to emotional cues in fatigued states, a finding that has important implications for clinicians dealing with fatigue and emotional work such as therapists.

Irum et al. review the recent literature on the gut microbiome in depressive disorder. They note the central role of the vagus in the connection between the gut and central nervous system regulation of affect, as well as the positive and negative contributions of various prokaryotic genera to mood. Which are well-established findings in the literature (Foster et al., 2017). More recent findings linking the actions and interactions of selective serotonin re-uptake inhibitors with the vagus and the gut are also reviewed. The latter finding are particularly interesting given the fact that most of the serotonin in the body is located in the gut rather than in the brain, as well as the long established folk wisdom that we feel things in our gut, an observation that rings true to any of us who has been nauseated by anxiety. More importantly, it firms up a linkage between irritable bowel syndrome and affect that has been noted before, but whose mechanism had not been fully clarified until recently.

Wang and Yin reassess the theory behind cognitive reappraisal techniques in the therapeutic environment by introducing schema theory, and LeDoux and Pine (2016) dual-system theory to the equation. Cognitive reappraisal is an important tool in many psychotherapeutic approaches. The underlying assumption of many of these approaches is that by changing cognitive appraisals in the clinic we can change the maladaptive schema that might lead to mental illness. The authors note, correctly, that a substantial challenge to the success of these approaches is the difference in context and context cues in the daily life and an individual vs. the clinic or laboratory. Wang and Yin, in their reframing of cognitive reappraisal and schema hope to improve the success of these approaches by attending to underlying schema in a way that provides more rapid and durable cognitive and emotional change. Grossman and Avital review the literature supporting the idea that emotional and sensory regulation may be an important factor in ADHD. A number of clinicians have noted a linkage between childhood adversity and ADHD (e.g., Harris, 2018), and recent empirical studies have provided evidence of a link (Crouch et al., 2021). The authors argue that this link points to emotional dysregulation as having a role in the disorder, as stressed and traumatized children are likely to have to spend cognitive resources on self-regulating their internal states rather than on paying attention. Further they review the literature on methylphenidate and conclude that while it is an effective therapy for ADHD, research into sensory and emotional regulation oriented interventions could improve its effectiveness in children suffering from the disorder.

All in all a broad and interesting set of insights for 2022. Hopefully these will provide stimuli for many future insights into how emotion is understood for many years to come.

## Author contributions

RH: Writing–original draft, Writing–review and editing.
